# Diagnosis of Meningitis Caused by Pathogenic Microorganisms Using Magnetic Resonance Imaging: A Systematic Review

**DOI:** 10.29252/nirp.bcn.9.2.73

**Published:** 2018

**Authors:** Alia Saberi, Seyed-Ali Roudbary, Amirreza Ghayeghran, Samaneh Kazemi, Mozaffar Hosseininezhad

**Affiliations:** 1. Neurosciences Research Center, Department of Neurology, Pouursina Hospital, School of Medicine, Guilan University of Medical Sciences, Rasht, Iran.; 2. Department of Neurology, Poursina Hospital, School of Medicine, Guilan University of Medical Sciences, Rasht, Iran.; 3. Deputy of Research and Technology, Guilan University of Medical Sciences, Rasht, Iran.

**Keywords:** Magnetic resonance imaging, Meningitis, Bacterial

## Abstract

**Introduction:**

Bacterial meningitis is an acute infectious inflammation of the protective membranes covering the brain. Its early diagnosis is vital because of its high morbidity and mortality. It is mostly diagnosed by a gold standard diagnostic tool i.e. Cerebrospinal Fluid (CSF) analysis. However, it is sometimes difficult and or impossible to do this procedure and an alternative diagnostic tool is needed. Contrast enhanced magnetic resonance imaging can detect the pus or other changes in subarachnoid space. But our optimal aim is to use an imaging method without using contrast to be useable and available in more specific condition.

**Methods:**

This study aimed to survey the role of non-contrast Magnetic Resonance Imaging (MRI) in the diagnosis of the bacterial meningitis. MEDLINE/PubMed Central, Web of Science and Scopus were searched without time period and language limitation until March 2017. We found 6410 papers in our initial search. After assessing the content of the papers based on Cochrane library guidelines and inclusion/exclusion criteria, 6 relevant studies were included in the systematic review. All of included studies were observational studies.

**Results:**

MRI studies demonstrated that Fluid Attenuation Inversion Recovery (FLAIR) and Diffusion-Weighted Image (DWI) MR imaging among all MRI modalities can detect some abnormalities compatible with bacterial meningitis. FLAIR and DWI-MR imaging are potentially useful to diagnose bacterial meningitis and can be used in emergent condition in which bacterial meningitis is highly suspicious and the other diagnostic tools are not available or feasible.

## 1. Introduction

Bacterial meningitis as a Central Nervous System (CNS) infection is a life-threatening disease. Pyogenic meningitis has a substantial risk of neurologic squeal leading to poor prognosis (Abe, Takayama, Yamashita, Noguchi, & Sagoh, 2002). In some instances, in spite of successful use of antibiotics, major neurologic complications occur (Jan et al., 2003). Meningitis is not frequent (1% of primary hospital admissions and 2% of nosocomially acquired infections), but it requires prompt diagnosis and specific treatment because of its high mortality and morbidity (Griffin, 1997; Kanamalla, Ibarra, & Jinkins, 2000; Johnson & Hayman, 2000). Different pathogens cause CNS infection and bacterial meningitis. Overall, Streptococcus pneumoniae is the most prevalent cause and the others, including Neisseria meningitidis, group B Streptococcus and Listeria monocytogenes have lower frequencies in order (Leedom & Underman, 2000; Schuchat et al., 1999).

Diagnosis of meningitis is based on clinical and laboratory findings like Cerebral Spinal Fluid (CSF) analysis as a gold standard method (Lummel et al., 2016). In this regard, nothing could replace CSF analysis. And even if in some cases, it could be difficult to obtain cerebrospinal fluid, it is always possible and necessary to retrieve these samples by other means such as radio- or ultrasound-guided lumbar puncture. But to save the time and when another paraclinical method is available, it is useful to start treatment and save the patients’ life. Although antibiotic treatment changes the pattern of CSF, delaying in treatment will result in high rate of mortality and morbidity.

Different Magnetic Resonance Imaging (MRI) modalities with respect to different sequences include sequences of Fluid Attenuated Inversion Recovery (FLAIR), Diffusion-Weighted Imaging (DWI), and T1-weighted enhanced with gadolinium. They are invaluable imaging techniques for diagnosis of complications of meningitis such as empyema, abscess, vasculitis, and infarction. Nowadays MRI has been suggested for diagnosis of meningitis itself even without any complication. Of course the determined sensitivities in different studies are very variable. Lummel et al. believed that the different severity of inflammation and the etiology of meningitis would result in different sensitivity of MRI in diagnosis of meningitis (Lummel et al., 2016).

The clinical usefulness of selective tissue suppression using inversion recovery pulse sequences has been extensively documented in the literature. Recently, Fluid Attenuated Inversion Recovery (FLAIR) MR, Diffusion Weighted Imaging (DWI) and Diffusion Tensor Imaging (DTI) techniques have been advocated for imaging patients with acute meningitis (Melhem et al., 1997). The nullification of the high signal of CSF in FLAIR occurs by the inversion recovery pulse which comes an inversion time. The elevation in protein and cellular concentration decrease T1 relaxation time of CSF, thus changing its null point. This leads to hyperintensity in the affected CSF, which can easily be detected by FLAIR (Adams & Melhem, 1999).

Reduced water diffusion in DWI appears as increased signal intensity and in its related Apparent Diffusion Coefficient (ADC) maps as decreased signal intensity (Noguchi et al., 1999; Guo, Provenzale, Cruz, & Petrella, 2001), like what appears in intracerebral abscess (Kim et al., 1998; Noguchi et al., 1999). There are different theories which explain the hypersignality of the CSF space seen by DWI in meningitis which are described as following (Abe et al., 2002). Diffusion Tensor Imaging (DTI) is one of the MRI modalities which shows the disturbance of white matter integrity based on the change in diffusion anisotropy. Although expensive, it can be used along with other non-contrast MRI modalities in assessing meningitis, because the periventricular white matter seems to be influenced by change in CSF composition (Malik et al., 2008). In this study, we will discuss Fluid Attenuated Inversion Recovery (FLAIR) MR, Diffusion Weighted Imaging (DWI) and Diffusion Tensor Imaging (DTI) techniques in diagnosis of acute meningitis.

## 2. Methods

### 2.1. Search strategy

Preferred Reporting Items for Systematic reviews and Meta-Analysis (PRISMA) was our guideline in this review (Moher et al., 2009). Searching was carried out in databases of PubMed, Scopus, and Web of Science. There was not any language, type of document and initial time limitation, and the articles published until March 2017 were studied. The keywords searched in the PubMed, Scopus, and Web of Science databases included “meningitis,” “magnetic resonance imaging,” “MR imaging,” and “MRI”. The search terms with similar meanings were combined using the OR logic, and the search terms were coupled using the AND logic.

The exclusion criteria were articles on: MRI with contrast (Gadolinium); Animal model; Presence of the focal lesion in the brain such as cerebritis, abscess, encephalitis, and so on; Immonocompromised host such as HIV^+^ (Human Immunodeficiency Virus) OR VDRL + (The Venereal Disease Research Laboratory test); Patients after surgery; Pachymeningitis; Meningoencephalitis; Cerebral malaria; and Meningitis of tuberculosis. In order to select the appropriate articles we assessed the abstracts of the searched articles or only their titles. If necessary, the full text of articles were reviewed, too. Reviewing was performed by two reviewers. After full assessment of the articles, the eligible articles were included in study.

### 2.2. Assessing the quality of articles

The STARD (Standards for Reporting of Diagnostic Accuracy) was used in this review, which is a quality standard for diagnosis of the completeness and transparency of studies (Scales et al., 2008).

### 2.3. Methods of data extraction

After screening available resources and databases, the selected articles were reviewed to extract their data uniformly.

## 3. Results

**Figure 1.**
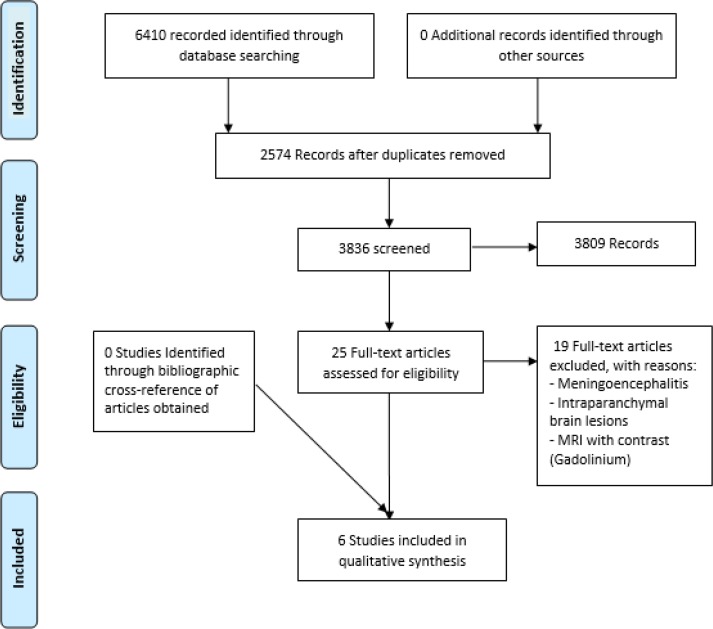
PRISMA 2009 Flowchart for the included studies of diagnosis of Meningitis caused by pathogenic microorganisms using MRI

After assessing the titles and abstracts of the articles and deleting duplicate articles, 3836 articles were remained. After omitting 3809 unrelated papers, 25 full texts were reviewed in term of eligibility. Considering the exclusion criteria during studying of their full texts, 6 articles were remained in this review. PRISMA Flow Diagram of this review summarizes the article acquisition (Figure 1). The selected articles (n=6) included three review articles and three original papers which their characteristics are listed in Table 1.

**Table 1.** General features and the results of the studies

**Author, Year, Country**	**Study Period**	**Study Type**	**Number of Participants**	**Age**	**Case Group**	**Control Group**	**Exclusion Criteria**	**Imaging Modality**										**Main Results (Concern Diagnosis of Meningitis)**
Ferreira et al., 2005, Brazil	-	Review	-	-	Diffuse pyogenic infections of the CNS (meningitis)	-	Aseptic (usually viral), Chronic (fungus, parasites)	Brain MRI/T1WI Brain MRI/Axial T1WI post contrast Brain MRI/Axial FLAIR Brain MRI/Axial FLAIR post contrast Brain MRI/Axial T2WI Brain MRI/DWI	The elevation of protein levels in Subarachnoid Space (SAS) leads to decrease in T1 relaxation time and increase the signal IN SAS in FLAIR sequence. Therefore, FLAIR sequences are very sensitive to detect pyogenic meningitis
Hazany et al., 2014, Los Angeles, CA	-	Review	-	-	Adult patients with infectious meningitis	-	-	Brain MRI/pre contrast T2-FLAIR	Increase CSF intensity in the sulci and basal cisterns may be seen on Fluid Attenuated Inversion Recovery (FLAIR) images in infectious meningitis
Hughes et al., 2010, UK	-	Review	-	-	Patients with acute bacterial meningitis and its complications (but we report only the results of pure bacterial meningitis)	-	-	Brain MRI/Axial FLAIR	In FLAIR sequences of case of meningitis, the signal of CSF in SAS is more than ventricular CSF, due to higher protein level caused by the leaky capillaries On FLAIR and T2 weighted images, the subcortical white matter can appear hypointense in areas underlying intense meningeal inflammation Both above mentioned signs are non-specific
Lummel et al., 2014, Germany	2007– 2012	Original	136 Patients, (55 males and 81 females) 75 patients with MRI acquired (44 females and 31 males)	16–81 years- Mean age = 53.0 years	Patients with bacterial meningitis (confirmed by CSF culture and/or clinical symptoms, in combination with typical CSF parameters changes)	-	-	Brain MRI/DWI (n=71) Brain MRI/FLAIR (n=49) Brain MRI/contrast-enhanced T1-weighted (n=63) Brain MRI/T2-weighted (n=63) Brain MRI/T2* (n=15) (1.5 Tesla)	62 Patients from 75 patients showed meningitis associated intracranial MRI abnormalities: Signal alterations were most frequently evident on FLAIR (79.6% in total) and DWI (67.6% in total). Which determines the similar frequency of FLAIR and DWI sequences abnormalities in meningitis and similar sensitivity for its diagnosis. Also an assessment was made on the radiologic difference based on bacterial specimen and resulted in intraventricular and/or sulcal diffusion restrictions were significantly more often in streptococcal meningitis than other agents.
McKinney et al., 2006, USA	-	Original	15 Patients	-	Patients with a high clinical suspicion for meningitis (it was confirmed by direct biopsy in 3) or CSF culture in 4 patients, one patient having septic emboli)	-	-	Fat suppression SPIR (spectral inversion recovery) and subtraction FLAIR imaging	Prior to intravenous contrast administration, 12 of the 22 examinations showed abnormal sulcal hyperintensity on precontrast SPIR/FLAIR images. The sensitivity and specificity of the noncontrast SPIR/FLAIR imaging for proven meningitis were 71% (5.7) and 75% (6.8) respectively.
Malik et al., 2008, India	2007	Original	14 Babies (9 males and 5 females)	Mean age. 13.28 days	Neonates with confirmed bacterial meningitis by CSF analysis, meningeal enhancement on gadolinium enhanced of meningeal layer in MRI (8 cases confirmed by CSF culture)	7 Age/sex matched controls with normal neurological examination (mean age = 12.85 days)	-	The Diffusion Tensor Imaging (DTI): (At the time of diagnosis and after 3 weeks of antibiotic treatment) The Fractional Anisotropy (FA) and Mean Diffusivity (MD) values were measured on the periventricular white matter of Frontal (FWM), Parietal (PWM), Occipital (OWM), and Temporal (TWM) lobes	MR DTI shows the extent of periventricular white matter injury in neonatal meningitis better than conventional MRI. Also decreased FA values in periventricular white matter is associated with high morbidity and mortality. But mean diffusivity (MD) value didn’t have any role in determining the outcome of meningitis.

## 4. Discussion

### 4.1. Fluid Attenuation Inversion Recovery (FLAIR)

Some authors believe that FALIR is a sensitive technique for detecting elevation of CSF protein and cellular content. In meningitis, elevations in protein and cellular concentrations decrease Tl relaxation time of CSF. Thus, changing its null point. This leads to hyperintensity in the affected CSF, which is easily detected on FLAIR (Adams & Melhem, 1999; Hazany et al., 2014). Some other authors found that high level protein conditions, as a non-specific finding for meningitis, might be seen in several other diseases and even in normal conditions such as slow arterial flow, disruption of blood-brain barrier, paramagnetic effects of supplemental O_2_ administration and also deposition of free radicals (Maeda et al., 2003; Taoka et al., 2001; Lee et al., 2002). Hughes et al. (2010) showed that in inflammatory condition the CSF in the Subarachnoid Spaces (SAS) can be of higher signal intensity than ventricular fluid, because of leaky capillaries which increase the level of proteins in that fluid (Hughes et al., 2010). However it is quite a non-specific sign and similar signal alteration can be caused in the other conditions such as carcinomatous meningitis, subarachnoid hemorrhage, oxygen and some drugs administration such as propofol (Castillo, 2004).

Another interesting finding of meningitis on FLAIR and T2 weighted sequences is hypointensity of subcortical white matter underlying intense meningeal inflammation (Lee et al., 2002) by unknown reason. Disordered oxygen free radical transport is the probable mechanism which can explain this signal alteration. It is also a non-specific finding (Hughes et al., 2010).

### 4.2. FLAIR vs. contrast enhanced T1 weighted MRI

Also FLAIR image of meningitis has been introduced superior to contrast enhanced T1 weighted MRI. SAS enhancement by contrast administered T1-weighted MRI in meningitis sometimes is difficult to be differentiated from normal prominent enhancing vascular structures. Non-contrast enhanced FLAIR can make such differentiation and resolve this problem. Additionally sometimes the swelling of the brain or widening of the extra-axial CSF spaces may be evident in meningitis which can be evident by non-contrast MRI sequences (Castillo et al., 1994; Hughes et al., 2010). Also, it is helpful in the condition in which the contrast cannot be used like glomerular filtration rate of lower than 30 mL/min/1.73 m^3^.

### 4.3. Post-contrast T1WI and post-contrast FLAIR

**Figure 2.**
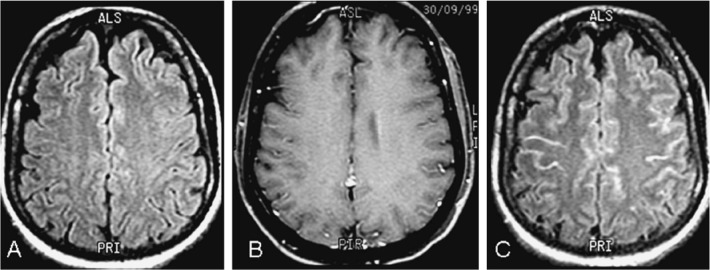
Patient with CSF analysis positive for meningitis A: Axial FLAIR imaging without contrast shows no abnormality. B: Axial post-contrast T1W1 without leptomeningeal enhancement. C: Axial post-contrast FLAIR imaging. Note in this example how this sequence shows significant leptomeningeal enhancement (arrows) comparing to the T1W1 (Ferreira et al., 2005).

Although our research is about non-contrast MRI modalities but it is interesting to mention the study by Ferreira et al. which compared post-contrast T1WI and post-contrast FLAIR images. Some investigators (Mathews et al., 1999) believed that post-contrast FLAIR imaging is more sensitive in detecting SAS diseases than T1WI post gadolinium and vice versa (Kamran, Bener, Alper, & Bakshi, 2004; Galassi et al., 2005). Advocates of the post-contrast FLAIR mention these main advantages: In FLAIR sequence with contrast, vessels enhancement with slow-flowing blood does not appear. But enhancement of vessels is visible on T1-weighted with contrast which is similar to meningeal enhancement; Gadolinium enhancement of the SAS is better visible by FLAIR compared with T1 weight sequence (Figure 2) (Singer et al., 1998; Mathews et al., 1999; Mamourian et al., 2000; Jackson & Hayman, 2000).

### 4.4. SPIR and fat suppression FLAIR

**Figure 3.**
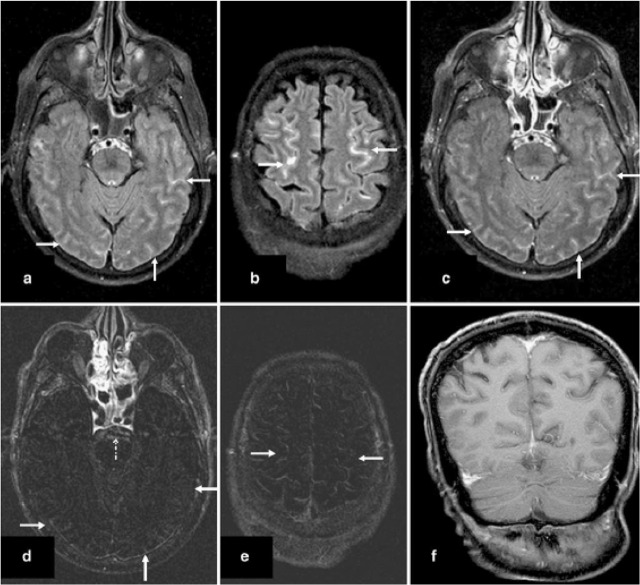
Sulcal hyperintensity in a patient with meningitis was not enhanced after subtraction of fat-suppressed SPIR/FLAIR images. Diffuse hyperintensity (arrows) in the cerebral sulci on FLAIR images before (a, b) and after (c) contrast (3-tesla). Subtraction images do not show the meningeal enhancement (d, e; arrows). On T1-W image with contrast, no meningeal enhancement is seen (f). After subtraction, the artifact of CSF flow in the prepontine cistern has been omitted (d, dashed arrow) (McKinney et al., 2006).

Fat suppression SPIR (Spectral Inversion Recovery) and subtraction FLAIR imaging (Jackson et al., 1998; Jackson et al., 1999) deliver better fat suppression rather than T1-weighted (T1-W) sequence, since the selected inversion time relates to the null point (T1 recovery time) of fat rather than the precessional frequency (Westbrook, Kaut Roth, & Talbot, 2005). It was documented in one study by McKinney et al. that sensitivity and specificity of the sequences of non-contrast SPIR/FLAIR imaging in the initial scan of patients with proven meningitis were 71% and 75%, respectively (Figure 3 a, b) (McKinney, Palmer, Short, Lucato, & Truwit,, 2006).

### 4.5. Diffusion Weighted Imaging (DWI)

**Figure 4.**
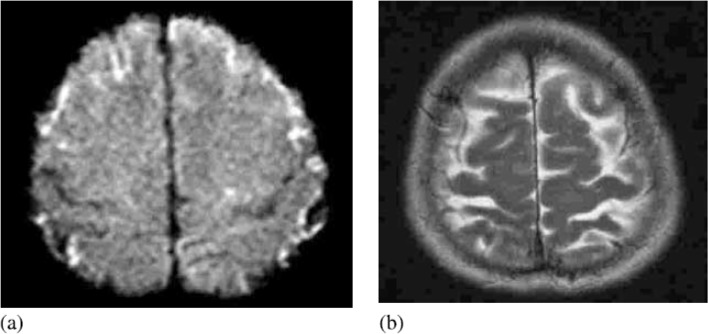
MRI on admission (a) Diffusion-weighted imaging shows diffusely scattered high-intensity lesions, mainly over the surface of the brain. (b) T2-weighted imaging shows no evidence of infarction where DWI showed high intensity (Abe et al., 2002).

DWI is a MRI sequence which has been introduced as a valuable tool for the diagnosis of acute meningitis and its complications (Noguchi et al., 1999; Guo et al., 2001). In this sequence, pus in the ventricles, especially lateral ventricles and in the cortical sulci appears hyperintense with high sensitivity (Rana, Albayram, Lin, & Yousem, 2002; Fujikawa, Tsuchiya, Honya, & Nitatori, 2006; Han et al., 2007). In a case of bacterial meningitis presented by Abe et al., MRI of the brain was normal in a T2-weighted sequence (T2WI), but suggested possible hyperintense lesions along the convexities in DWI (Figure 4). Four days after admission, follow-up DWI confirmed hyperintense lesions both along the convexities and Sylvian fissure, and also in the third and the fourth ventricle; T2WI continued to appear normal. Enhancement by gadolinium contrast administration highlighted the meningeal exudate as well as the lesions throughout the convexities and Sylvian fissure that were seen by DWI (Abe et al., 2002).

DWI is highly sensitive in acute ischemic infarction. Considering the macro- and micro-infarction due to septic emboli and or vasculitis as complication of the bacterial meningitis which is evident in nearly 20% of patients (Lummel et al., 2016), another advantage of this sequence of MRI could be also its value in prognostic approach.

Thus, Abe et al. described two theories for visualized hyperintensity in DWI: The contents of purulent meningitis is responsible for the high signal intensity; and Small-vessel vasculitis with cortical infarcts would seem as the basis for the patient’s hyperintense lesions in DWI (Abe et al., 2002).

For confirmation of these two theories, Ebisu et al. (1996) and Desprechins et al. (1999) reported patients with brain abscess who showed high signal intensity in the abscess cavity in DWI. To better understand these findings, Ebisu subsequently obtained DWI of purulent contents aspirated from the abscess. Again high signal intensity was evident, leading the authors to conclude that abscess contents were responsible for the high signal intensity in DWI in vivo. These findings are probably related to restriction of water molecules movement in a viscous compound, including proteins, inflammatory cells, and infection microorganism. Additionally, water molecules can be limited in their translational movement by binding to carboxyl, hydroxyl, and amino groups on surfaces of macromolecules in the abscess (Rusakov & Kullmann, 1998).

In autopsy cases of tuberculosis and Haemophilus meningitis, cerebral arteriopathy has been attributed to purulent leptomeningeal exudates (Lyons & Leeds, 1967; Yamashita et al., 1985; Greitz, 1964), particularly at the base of brain but also spreading along sulci of the convexity in proximity to which become inflamed (Yamashita et al., 1985; Greitz, 1964). Accordingly the MRI findings in the case presented by Abe et al. may represent purulent leptomeningeal exudates similar in character to the highly intense lesions seen with DWI in patients with brain abscess. In fact, DWI showed diminishing signal changes as CSF findings improved. These findings may strongly support this possibility. Somewhat less likely in their case is a possibility that highly intense lesions in DWI may reflect ischemia, as seen in acute infarction. Greitz first reported narrowing of the arteries at the base of the brain in tuberculous meningitis (Greitz, 1964). Subsequently, Lyons and Leeds described occurrence of purulent meningitis associated with vasospasm, stenosis, and occlusion (Lyons & Leeds, 1967) probably related to the inflammation of vessel walls (Yamashita et al., 1985). Vasculitis predominately affecting small vessels can produce small areas of active ischemic change not visible in conventional MRIs but detectable by DWI (Yuh et al., 1998). In Abe et al. case, follow-up MRI showed infarct-like lesions in T2WI that correlated with the highly intense lesions seen by DWI. Thus, small-vessel vasculitis with cortical infarcts would seem relatively unlikely as the basis for this patient’s hyperintense lesions in DWI (Abe et al., 2002).

**Figure 5.**
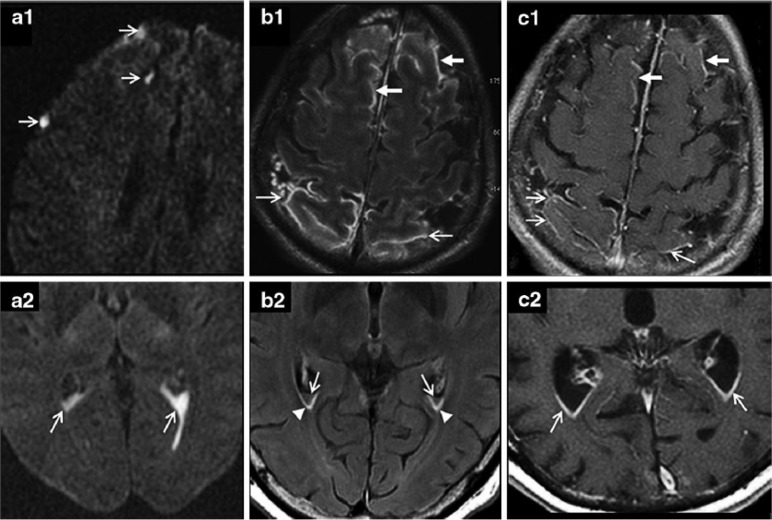
Diffusion-Weighted Imaging (DWI) (a), Fluid Attenuated Inversion Recovery (FLAIR) (b), and contrast-enhanced T1-weighted images and (c), in acute bacterial meningitis On DWI, pus is visualized in sulci (a1, thin arrows) and intra-ventricles (a2, thin arrows) as diffusion restriction. On FLAIR sequence increased signal is seen in sulci of the frontal (b1, thick arrows) and parietal (b1, thin arrows) lobes, and intra-ventricles (b2, thin arrows) and sub-ependymal space because of protein enrichment (b2, arrowheads). Both leptomeningeal and ependymal gadolinium - enhancement in T1sequence in frontal (c1, thick arrows) and parietal (c1, thin arrows) lobes (Lummel et al., 2014).

Lummel et al. determined the sensitivity of DWI and FLAIR for detecting meningitis, 67.6% and 79.6%, respectively. Table 2 presents the presence and anatomical location of signal changes in the different MRI sequences. Sulcal abnormities in DWI and FLAIR were evident in 18 out of 71(25.4%) patients and 11 out of 49(22.4%) patients, respectively and cortical abnormities in 9 out of 71(12.7%) patients and 7 out of 49(14.3%) patients, respectively (Figure 5). Hyperintense signals of FLAIR sequences within the sulcal and or intraventricular space in meningitis may be due to protein-enrichment of the CSF. Areas with diffusion restriction within the CSF spaces in DWI were interpreted as pus. Also there is similar frequency (near 50%) of FLAIR and DWI sequences abnormalities in meningitis and similar sensitivity for its diagnosis (Lummel et al., 2014).

**Table 2.** Signal change in different MRI sequences in bacterial meningitis (Lummel et al., 2014)

Sequence Available/Total Patients, n (%)	All Sequences 75.75	DWI 71.75	FLAIR 49.75	T2 63.75	T2* 15.75	T1+ 63.75
All localizations	62(82.7)	48(67.6)	39(79.6)	31(49.2)	0(0)	32(50.8)
Intraventricle	41(54.7)	35(49.2)	24(49)	18(28.6)	0(0)	N.A.
Sulcus	22(29.3)	18(25.4)	11(22.4)	4(6.3)	0(0)	N.A.
Cortex	15(20)	9(12.7)	7(14.3)	7(11.1)	0(0)	4(6.3)
White matter	20(26.7)	12(16.9)	10(20.4)	16(25.4)	0(0)	5(7.9)
(Sub) ependymum	N.A.	N.A.	22(44.9)	N.A.	N.A.	18(28.6)
Leptomeninge	N.A.	N.A.	N.A.	N.A.	N.A.	17(27)
Dura	N.A.	N.A.	N.A.	N.A.	N.A.	6(9.5)

DWI: Diffusion-Weighted Imaging; FLAIR: Fluid Attenuated Inversion Recovery; +: With contrast; N: Number; N.A.: Not Analyzed; T2*: Weighted gradient echosequence

Another interesting finding in Lummel et al. study was the specificity of DWI with respect to the causative pathogen. So that, the patients with streptococcal meningitis showed restriction pattern in ventricles and sulci more frequent than patients with other types of meningitis (85.3% vs. 32.4%) (P<0.0001) (Lummel et al., 2014). Whereas in the other studies it was claimed that simple magnetic resonance imaging is usually nonspecific for the pathogens, because of the similarity of the brain response to different bacterial species (Hazany et al., 2014).

### 4.6. Diffusion Tensor Imaging (DTI)

**Figure 6.**
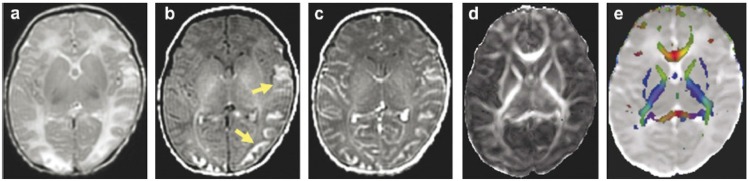
A case with clinical and para-clinical confirmed meningitis. There are signal alteration in the bilateral cortex, subcortex, and periventricular white matter of occipital area (arrow) and left temporal cortex on T2-weighted (a)/T1-weighted (b) images, respectively. The increased signal in cortex (b) is consistent with cortical laminar necrosis. Meningeal enhancement in gadolinium- T1-weighted (c). Reduced FA values in periventricular white matter in FA map (d). The abnormalities visible on T1 sequence are not demonstrated on FA map (d) and color coded FA (e) fused with MD (Malik et al., 2008).

Diffusion Tensor Imaging (DTI) is one of the MRI modalities which assesses the integrity of brain structure using diffusion anisotropy. Malik et al. in a study determined the change in periventricular white matter signal due to damage of its structure and integrity in bacterial meningitis by DTI (Figure 6) (Malik et al., 2008). Also they studied the outcome of patients by this technique of MRI by determining the FA (fraction of anisotropy) values of periventricular white matter at the time of base and follow-up study (Tables 3 and 4).

**Table 3.** Fractional Anisotropy (FA) values of the periventricular white matter of different regions of patients with meningitis and control group at the base of study (Malik et al., 2008)

**Region of White Matter**		**Control (a) (n=7)**	**Normal Outcome (b) (n=7)**	**Abnormal Outcome (c) (n=7)**	**P**
Frontal	Right	0.11±0.03	0.09±0.02	0.06±0.01	pab=0.27, pac=0.03, pbc=0.07
	Left	0.12±0.03	0.09±0.02	0.06±0.03	pab=0.12, pac=0.03, pbc=0.09
Parietal	Right	0.13±0.03	0.09±0.03	0.07±0.03	pab=0.03, pac=0.01, pbc=0.46
	Left	0.10±0.01	0.10±0.03	0.07±0.03	pab=0.99, pac=0.07, pbc=0.17
Occipital	Right	0.15±0.03	0.10±0.03	0.07±0.02	pab=0.04, pac=0.00, pbc=0.02
	Left	0.16±0.04	0.11±0.01	0.07±0.02	pab=0.02, pac=0.00, pbc=0.00
Temporal	Right	0.13±0.01	0.12±0.02	0.07±0.03	pab=0.45, pac=0.00, pbc=0.01
	Left	0.14±0.03	0.12±0.04	0.07±0.02	pab=0.16, pac=0.00, pbc=0.06

b: No neurological abnormality (clinical and imaging); c: Demise or neurological abnormality (clinical and imaging)

**Table 4.** Fractional Anisotropy (FA) values of the periventricular white matter of different regions of patients with meningitis and control group at the time of follow-up study (Malik et al., 2008)

**Region of White Matter**		**Control (a) (n=7)**	**Normal Outcome (b) (n=7)**	**P**
Frontal	Right	0.12±0.03	0.09±0.02	0.12
	Left	0.11±0.04	0.09±0.03	0.19
Parietal	Right	0.14±0.02	0.09±0.03	0.23
	Left	0.12±0.02	0.10±0.03	0.11
Occipital	Right	0.16±0.05	0.11±0.01	0.03
	Left	0.14±0.04	0.10±0.01	0.04
Temporal	Right	0.13±0.01	0.11±0.01	0.02
	Left	0.13±0.02	0.14±0.03	0.28

b: No neurological abnormality (clinical and imaging)

The FA in periventricular white matter decreases in meningitis due to the vulnerability of this anatomic location to change of CSF composition which appears normal in other routine MRI sequences. This change may be the result of inflammation of vessels which make them prone to thrombosis and consequently producing ischemic infarct and decreased blood perfusion in that area. Also, periventricular neuronal and glial injury may occur due to the release of free radicals and proteases, and toxic cytokines in response to inflammation (Nau & Bruck, 2002). Also decreased FA values were obvious on follow-up study in some patients. This could be the result of oligodendroglial cells damage and deficient myelination process.

They assessed the valuable role of DTI in determining outcome in meningitis. They suggested the morbidity and mortality of neonatal meningitis can be estimated by determining FA values in periventricular white matter. But mean diffusivity (MD) value lacked any role in determining the outcome of meningitis (Malik et al., 2008). So the use of DTI in meningitis may have a prognostic value.

FLAIR and DWI-MR imaging are potentially useful to diagnose the conditions such as bacterial meningitis that SAS or CSF contain high cellular or protein materials. Also it can be used in emergent condition in which bacterial meningitis is highly suspicious and the other diagnostic tools are not available or feasible. CSF analysis is the gold standard of diagnosis of meningitis and it is necessary to be done as soon as possible by every aiding tools such as radio- or ultrasound-guided lumbar puncture. But because of the life threatening nature of meningitis and in order to save the time, every available tool is justified to save the patients’ life. Regarding this fact, FLAIR and DWI sequences of brain MRI can help the physician to diagnose meningitis. In order to better clarity this systematic review, we need a meta-analysis.
